# Reduction of Anxiety-Related Symptoms Using Low-Intensity Ultrasound Neuromodulation on the Auricular Branch of the Vagus Nerve: Preliminary Study

**DOI:** 10.2196/69770

**Published:** 2025-05-01

**Authors:** Izzy Kohler, Jon Hacker, Ethan Martin

**Affiliations:** 1 NeurGear, 295 Warrington Dr, Rochester, NY, 14618, United States, 1 5853718308

**Keywords:** low-intensity focused ultrasound, auricular branch of the vagus nerve, anxiety, depression, posttraumatic stress disorder

## Abstract

**Background:**

Neuromodulation of the auricular branch of the vagus nerve using low-intensity focused ultrasound (LIFU) is an emerging mode of treatment for anxiety that could provide a complementary or alternative treatment modality for individuals who are refractory to conventional interventions. The proposed benefits of this technology have been largely unexamined with clinical populations. Further research is required to understand its clinical potential and use in improving and managing moderate to severe symptoms.

**Objectives:**

The aim of this study was to do a preliminary investigation into the efficacy, safety, and usability of the wearable headset that delivers LIFU to the auricular branch of the vagus nerve for the purpose of alleviating anxiety disorder symptoms.

**Methods:**

This study was a pre-post intervention study design for which we recruited 28 participants with a Beck Anxiety Inventory score of 16 points or greater. Participants completed 5 minutes of treatment daily consisting of LIFU neuromodulation delivered to the auricular branch of the vagus nerve. Participants did this for a period of 4 weeks. Assessments of anxiety symptom severity (Beck Anxiety Inventory), depression symptom severity (Beck Depression Inventory), posttraumatic stress disorder symptom severity (Post Traumatic Stress Disorder Checklist for the *Diagnostic and Statistical Manual of Mental Disorders* [Fifth Edition]), and sleep quality (Pittsburgh Sleep Quality Index) were taken prior to starting treatment and weekly for 4 weeks of treatment. Usability and safety were also assessed using an exit questionnaire and adverse event logging.

**Results:**

After completing 4 weeks of LIFU neuromodulation to the auricular branch of the vagus nerve, the average Beck Anxiety Inventory score decreased by 14.9 (SD 10.6) points (Cohen *d*=1.06; *P*<.001), the average Beck Depression Inventory score decreased by 10.3 (SD 7.8) points (Cohen *d*=0.81; *P*<.001), the average Post Traumatic Stress Disorder Checklist for the *Diagnostic and Statistical Manual of Mental Disorders* (Fifth Edition) score decreased by 20.0 (SD 20.5) points (Cohen *d*=0.94; *P*<.001), and the average Pittsburgh Sleep Quality Index score decreased by 2.2 (SD 3.1) points (Cohen *d*=0.65; *P*=.001). On the exit questionnaire, participants rated the treatment highly for ease of use, effectiveness, and worthiness of the time invested. Only 1 adverse event was reported throughout the entire trial, which was mild and temporary.

**Conclusions:**

This preliminary study provided justification for further research into the efficacy, safety, and feasibility of using LIFU to modulate the auricular branch of the vagus nerve and reduce the symptoms of anxiety, depression, and posttraumatic stress disorder.

## Introduction

Anxiety is the “anticipation of real or imagined future threat or danger” [1], which manifests itself with a mix of emotional signals, such as hyperarousal and panic, and physiological ones, including increased heart rate, shortness of breath, sweating, and chest pain [2]. The emotional and physiological responses experienced with anxiety result from the activation of the hypothalamus, which engages the sympathetic nervous system (SNS) [3,4]. This sympathetic activation is adaptive in short bursts and enables us to handle threats and stressors, but in anxiety disorders, the SNS may be overly sensitive or chronically activated, leading to distress and health challenges over time [5,6]. Clinically significant anxiety symptoms are disproportionate to the future threat, endure after it has passed, and cause substantial distress or incapacitation [1,7]. The etiology of anxiety disorders is complex, with heritability ranging from 30% to 67% depending on the research study and anxiety disorder type [1]. However, trauma, chronic stress, and other environmental factors play an important role in the development of maladaptive anxiety [7].

The complex etiology of anxiety opens opportunities for intervention at multiple points in the course of the illness from a variety of disciplines. There are also several multidisciplinary approaches that offer a more holistic care plan. The primary goal of preventative strategies is to lower the risk of developing disordered anxiety responses prior to onset. Preventative psychoeducational interventions for adolescents and adults have been shown to reduce the risk of anxiety onset [8] with small to moderate effect sizes [1,8]; however, studies of these interventions tend to end their follow-ups after only 9 months, so the long-term stability of their benefits after intervention completion is still in question [1]. Once an active anxiety disorder has developed, psychotherapeutic treatments for it range in intensity from self-guided programs to highly intense weekly sessions with a licensed therapist. Self-guided treatments derived from evidence-based psychotherapies are more effective than active controls but show smaller effect sizes than therapist-guided programs [9]. Cognitive behavioral therapy is widely considered to be the gold standard for anxiety disorder treatment, particularly in adults, although Haller et al [10] found mindfulness-based cognitive therapy and acceptance and commitment therapy to be similar in efficacy. In recent years, virtual psychotherapy modalities have emerged as a compromise that balances the convenience of self-help approaches and the rigor and guidance of a traditional in-person therapy session. Thus, recent advances in telehealth have paved the way for approaches that afford convenience and accessibility without a loss of efficacy [11,12].

Pharmacotherapy is similar in efficacy to psychotherapy, and both pharmacotherapy and psychotherapy are considered first-line treatments for anxiety disorders in most standard care plans [1]. Selective serotonin reuptake inhibitors, serotonin and norepinephrine reuptake inhibitors, benzodiazepines, antipsychotics, and β-blockers are all used to treat anxiety. Despite this wealth of options, anxiety disorders remain chronic and refractory to treatment in many individuals, with 15%‐40% achieving less than 50% remission in symptoms [13]. Studies of combinations of psychotherapeutic and pharmacological approaches to anxiety treatment are sparse, leaving confusion surrounding which combinations are most efficacious [1]. Taken as a whole, while current neurobiological and psychosocial treatment approaches to anxiety disorders are sufficient for a large portion of affected individuals, there is still a substantial proportion of patients who would benefit from additional treatment options.

Low-intensity focused ultrasound (LIFU) is an emerging mode of treatment for anxiety that could provide an alternative treatment modality. LIFU can stimulate or inhibit neural activity, depending on the parameters of the energy applied to neural tissue. Also referred to as acoustic neuromodulation, the use of LIFU to modulate the activity of neural structures is a promising method for noninvasive treatment of neurological disorders [14]. While the majority of investigations featuring LIFU neuromodulation have primarily focused on modulation of neural structures within the central nervous system, disorders affecting the peripheral nervous system stand to benefit from this powerful tool as well [15]. LIFU neuromodulation of the peripheral nervous system is accomplished through a nonthermal, noncavitation bioeffect produced by setting the parameters to the intermediate intensity range. At intensities between 1 and 200 W/cm^2^, ultrasound is able to noninvasively and reversibly enhance peripheral neural activities by activating low-threshold mechanosensitive nerve endings, opening mechanosensitive ion channels to evoke action potentials [15]. Ultrasound of intermediate intensity also enhances the neural activity of peripheral nerve axons, leading to increased nerve conduction velocities in both A- and C-type fibers, which is likely caused by mechanical gating of other ion channels [16]. In addition, enhanced neural activity could be attributed to a direct effect of acoustic radiation forces on the lipid-bilayer neural membrane. Plausible mechanisms for this include a transient capacitive current from rapid changes of local membrane capacitance and transmembrane pore formation to allow sodium and potassium ions to pass through [15,16].

The vagus nerve, also known as cranial nerve X, is the longest cranial nerve and its branches enable the organs to adjust to the demands of a person’s internal state and external environment. The vagus nerve is a primary component of the parasympathetic nervous system, which, paired with the SNS, constitutes the autonomic nervous system [4,17]. Normally, sympathetic and parasympathetic nerve pathways act synergistically to create a state of equilibrium appropriate to meet the demands of the current internal state and external challenges. Disruption of the balance of sympathetic and parasympathetic activity in favor of sympathetic activity is one indicator of anxiety disorders [4,18].

The many branches of the vagus nerve are increasingly seen as pathways for promoting or restoring health and ameliorating the physiologic unease that gives rise to anxiety and other negative mental states [19]. The vagus nerve operates bidirectionally, meaning states of homeostasis and calm can be induced from the bottom up or the top down. The brain can use cognitive strategies to dissipate states of bodily unease (top down) or activate vagal nerve pathways to create psychological comfort and a sense of safety (bottom up) [20]. In addition to its role in regulating the parasympathetic nervous system, the vagus nerve also projects to the amygdala and hippocampus, both of which are important to extinction learning techniques commonly used in the treatment of anxiety and posttraumatic stress disorder (PTSD) [21,22]. Stimulation of the vagus nerve can downregulate sympathetic activity, restoring visceral order and psychological calm [23,24].

Early research into the clinical applications of vagus nerve stimulation (VNS) primarily centered on epilepsy and depression [17], but the vagus nerve is an attractive target for antianxiety therapies as well. In addition to its role in regulating the parasympathetic nervous system, the vagus nerve also projects to the amygdala and hippocampus, both of which are important to extinction learning techniques commonly used in the treatment of anxiety and PTSD [21,22]. Preliminary clinical studies have demonstrated VNS’s therapeutic applications to treatment-resistant anxiety disorders [23] and long COVID-19 symptoms [25]. Physiological changes as an effect of VNS are also well known in the literature. Wittbrodt et al [26] discovered that transcutaneous cervical VNS increased activation of the anterior cingulate and hippocampus during exposure to traumatic scripts. Lamb et al [27] found that transcutaneous auricular vagal nerve stimulation (taVNS) improved respiratory sinus arrhythmia and skin conductance during exposure to physical and emotional stress. Bremner et al [28] found that transcutaneous cervical VNS decreased inflammatory markers and sympathetic tone while increasing medial prefrontal function during exposure to trauma-specific and neutral stressors.

While VNS is traditionally done electrically, ultrasound’s noninvasiveness and specificity make it ideal for VNS [29]. Ultrasound has been successfully used for vagus nerve neuromodulation in rats [30] and for peripheral nerve [29] and suborgan [31] stimulation in humans. With a recent study showing the feasibility of transauricular VNS as an at-home intervention [20,22], transauricular ultrasound VNS has emerged as a noninvasive, yet potentially effective, at-home treatment for the management of anxiety symptoms. In response to this, we have developed a wearable headset with an ultrasound transducer that delivers LIFU to the auricular branch of the vagus nerve that can be used at home for treatment of anxiety symptoms. The purpose of this study was to do a preliminary investigation into the efficacy, safety, and usability of the wearable headset that delivers LIFU to the auricular branch of the vagus nerve for the purpose of alleviating the symptoms of anxiety. Because depression [32] and PTSD [33] frequently co-occur with anxiety, we also investigated the efficacy of transauricular ultrasound VNS for alleviating the symptoms of depression and PTSD in individuals with anxiety.

## Methods

### Study Design

This was a pre-post-intervention study in which all participants received the intervention daily, at home, for a period of 4 weeks. The clinical trial is registered at ClinicalTrials.gov [NCT06574971]. Informed consent was obtained from each of the 28 participants prior to screening. All activities were completed remotely and a ZenBud device with a user manual and participant instructions was shipped to each participant’s home. Participants completed 5 minutes of LIFU to the auricular branch of the vagus nerve each day using the ZenBud device. Treatment could be completed at any convenient time of day and did not have to be completed at the same time every day, as long as the treatment was completed within every 24-hour period. Assessments were completed on the web on the day before the first treatment session and then weekly. The final assessment was completed on the day of the final treatment after the final treatment session. The battery of assessments included 4 validated clinical outcome measures: Beck Anxiety Inventory (BAI), Beck Depression Inventory (BDI), PTSD Checklist for *Diagnostic and Statistical Manual of Mental Disorders* (Fifth Edition) (*DSM-V*) (PC5), and Pittsburgh Sleep Quality Index (PSQI). The details of these assessments are further described in the data collection section.

### Participant Recruitment

Adults in the United States were recruited through web-based social media advertising mentioning a study investigating a new treatment for anxiety disorders. Interested individuals filled out a study registration form containing only contact information and were then contacted by a member of the research team via email with further details of the study and a link to sign the informed consent. Upon completion of the informed consent, candidates were then screened for inclusion and exclusion criteria using web-based questionnaires. Interested individuals were included if they scored 16 or higher on the BAI, were older than 18 years, and did not have any additional conditions that were contraindications for VNS or ultrasound. Conditions that were contraindications for VNS included a history of vagotomy, heart arrhythmias, schizophrenia, or rapid cycling bipolar disorder. Conditions that were contraindications for ultrasound included presence of a pacemaker, pregnancy, active cancer, decreased sensation or open wounds in the ear, ear infection, or metal implants in or around the ear. A BAI score of 16 was chosen as the cutoff threshold because a score of 16 or higher in the BAI classifies an individual as having moderate to severe anxiety symptoms [34]. We did not exclude individuals who were receiving other treatments for their anxiety as long as the treatment was not initiated or ceased within the past month.

A total of 100 individuals completed the interest form, 63 signed the informed consent and were screened, and 28 were enrolled in the study. Each participant was assigned a unique identifier code so that participant information could be managed in a confidential manner throughout the study and the data could be deidentified upon completion of the study. Only the principal investigator and the study coordinator had access to the unique identifier code assignments.

### Ultrasound Device

**Figure 1.**
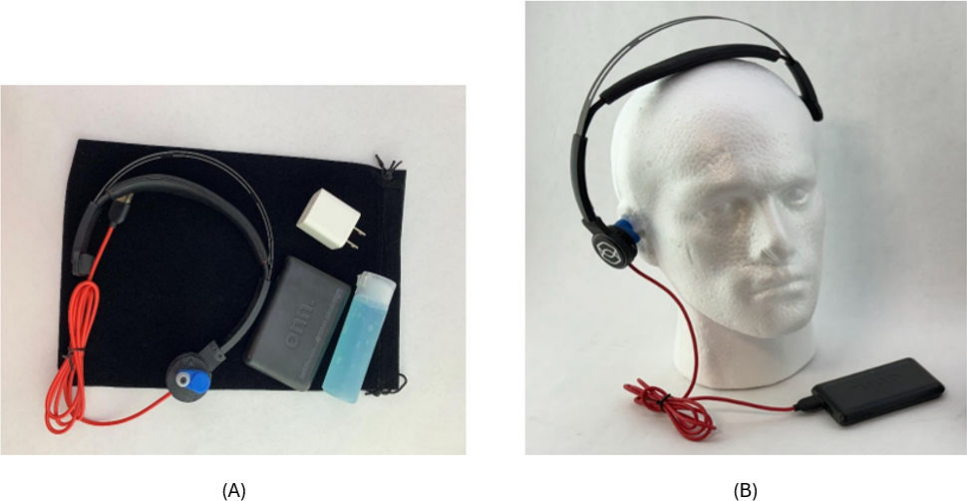
(A) The ZenBud headset, powerpack, power brick, and bottle of gel. The ultrasound transducer is located in the round earpiece on the right side of the headset. (B) The ZenBud device as depicted properly placed on a model human head.

ZenBud, the device used for this trial, is a proprietary Conformité Européenne–compliant over-the-ear wearable headset that was developed by NeurGear (Figure 1A and B). The ZenBud delivers LIFU to the auricular branch of the vagus nerve through several layers of skin. The ZenBud is designed to mimic a standard headset so that users can integrate the use of the device into their routine with minimal effort and discomfort. When the user plugs the ZenBud device into the battery pack it immediately turns on. There is a hardware limit in the circuitry so that the device shuts down after running for 29 minutes, limiting the duration of use. The ZenBud device specifications include a center frequency of 5.3 MHz, a pulse repetition frequency of 41 Hz, a duty cycle of 50%, and an average intensity of 1.03 MPa.

**Figure 2.**
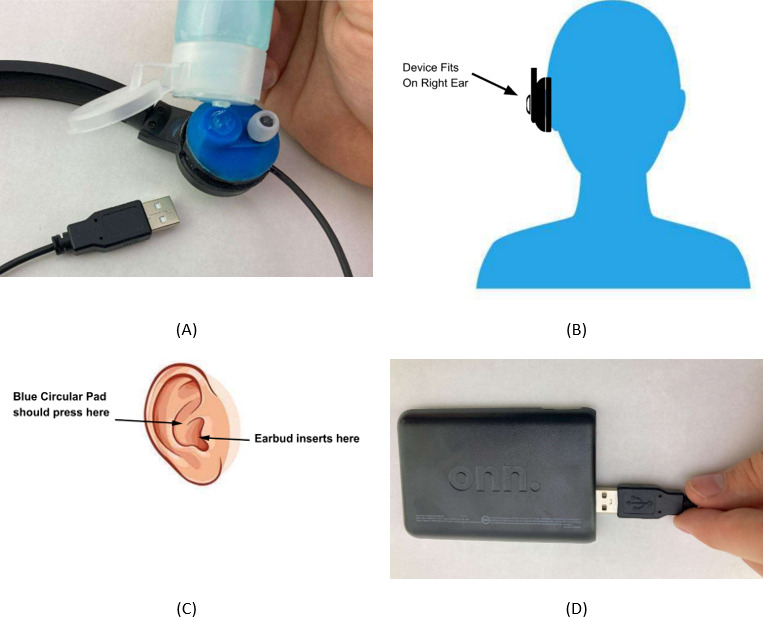
Images extracted from the ZenBud user manual depicting step-by-step setup and operation of the device. (A) Application of the ultrasonic gel. (B) Placement of the headset with the headset located over the right ear. (C) Correct placement of the headset on the ear. (D) Treatment is started upon inserting the USB cable into the battery pack.

A detailed instruction manual was provided in the package with every device. A copy of the manual is provided as Multimedia Appendix 1. The participants were instructed to use the device once a day for 5 minutes unless instructed otherwise by a health care professional. There were no stipulations set for the time of day that treatment could be completed and participants were free to choose a time that was convenient for them. For step-by-step set up and use, participants were instructed to apply a pea-sized amount of the aqua sonic gel to the blue part of the device located directly above the headset (Figure 2A), position the blue circular pad against the skin just above the ear canal (Figure 2C), adjust the headset until they felt a moderate pressure (without pain) just above the ear canal where the blue circular pad was positioned (Figure 2B), and begin stimulation by plugging the USB cable into the battery pack (Figure 2D). Once the headset is plugged into the battery pack the device starts working and a low humming noise can be heard. The manual instructs users to listen for the humming sound to indicate that the device is working properly.

### Data Collection

Assessments were done using a battery of 4 validated clinical outcome measures. These were taken on the day before the first treatment session, weekly, and on the day of the final treatment session after the final treatment session was completed. The following 4 clinical outcome measures were used.

#### Beck Anxiety Inventory

The BAI is a rating scale used to evaluate the severity of anxiety symptoms in individuals aged 17 years and older. It contains 21 self-report items that reflect common physiological symptoms of anxiety such as numbness or tingling, feeling hot, and trembling. Participants indicate how much they have been bothered by each symptom, from “not at all” to “severely,” using a 4-point Likert scale. The item scores are then summed, with possible scores ranging from 0 to 63. A total score of 0‐7 is classified as minimal anxiety, 8‐15 as mild, 16‐25 as moderate, and 26‐63 as severe [35,36]. The BAI has a Cronbach α value of 0.91, a good test-retest reliability (κ=0.65, 95% CI 0.61-0.69), and correlates moderately (Pearson *r*=0.51) with the revised Hamilton Anxiety Rating Scale (HAM-A) [34,35,37,38].

#### Beck Depression Inventory Version II

Depression and anxiety are highly comorbid, with 60% of patients with anxiety disorders also having depression [32]. Long-term activation of the stress response may explain this overlap [39], implying that inhibiting overactivation of the stress response may alleviate depressive symptoms in addition to anxiety and stress. The BDI-II is a valid and reliable self-report measure for depression that quantifies depressive symptoms over the last week [40]. For each of the 21 items, respondents are asked to choose the statement they most agree with out of a group of 4 choices. Each statement corresponds to a score ranging from 0 to 3 and total scores range from 0 to 63 [35,41,42]. The scores are classified as minimal depression (0‐13), mild depression (14-19), moderate depression (20-28), and severe depression (29-63) [38,41]. The BDI is positively correlated with the Hamilton Depression Rating Scale with a Pearson *r* of 0.71, showing good agreement. The test was also shown to have a high 1-week test-retest reliability (Pearson *r*=0.93), suggesting that it was not overly sensitive to daily variations in mood and high internal consistency (*α*=.91) [38,41].

#### PTSD Checklist for *DSM-V* (PCL-5)

While the *DSM-V* does not classify PTSD as an anxiety-related disorder, both PTSD and anxiety disorders involve dysregulation in neural structures dealing with fear, arousal, and anticipation of future threats [33]. Thus, there is reason to believe that VNS simulation could be beneficial for PTSD-related symptoms. The PCL-5 is a self-report questionnaire that helps assess the presence and severity of PTSD symptoms. The PCL-5 can be used to screen for PTSD, assist in making a provisional diagnosis, and monitor symptoms over time [43] . The measure asks participants to rate how much they were bothered by certain PTSD symptoms over the past month on a 5-point Likert scale ranging from “not at all” to “extremely” [44]. Total scores range from 0 to 60 and scores ranging from 31 to 33 are widely accepted as the cutoff for diagnosing PTSD [45]. In a systematic review of PCL-5 validation studies, Forkus et al [45] concluded that the full 20-item version showed good to excellent internal consistency across studies (Cronbach α values ranging from 0.83 to 0.97) and acceptable temporal stability (correlations ≥0.60) across time points within 1‐5 weeks of one another. Scores were also moderately to highly correlated with other measures of PTSD as well as measures of anxiety, depression, suicidal ideation, and sleep.

#### Pittsburgh Sleep Quality Index

Anxiety and sleep disturbance are frequently co-occurring [46] such that sleep disturbance is a *DSM-V* criterion for generalized anxiety disorder. Studies have found correlations between BAI scores and subjective sleep quality among college students [47], indicating that measuring sleep quality could provide insight into the burden of anxiety on well-being. The PSQI is a validated and widely used global measure of sleep quality [48,49]. It comprises 19 self-report items and 5 items to be reported by a sleeping partner, but the 19 self-report items are commonly used on their own in research contexts [50]. The different items call for responses in different formats (bedtimes, number of hours, Likert scales, etc), thus the instrument is scored with the use of 7 component scores that are summed for 1 total score ranging from 0 to 21 [48]. The original creators of the PSQI found that a score of 5 or greater differentiated between “good” and “poor” sleepers, with a sensitivity of 89.6% and a specificity of 86.5% [48]. Research since has generally supported the validity of this cutoff. Mollayeva et al [49] did a meta-analysis of the psychometric properties of the PSQI and found that it showed acceptable internal reliability for within-group comparisons across studies (Cronbach α values ranging from 0.70 to 0.83). They also found that intraclass correlations for PSQI scores across timepoints met the cutoffs for use in within-group comparisons (0.70 or greater) [49].

#### Exit Survey

In addition to the clinical outcome measures, participants also completed an exit survey on the final day of the trial. This survey asked questions regarding overall satisfaction with the treatment, impact on daily functioning and quality of life, ease of use, symptom improvement, side effects, and how quickly effects from the treatment were perceived to be felt. The purpose of this questionnaire was to provide further insight into the perceived experiences of the participants during the treatment period, which is important information for full and complete understanding of the treatment’s impact.

### Adverse Event Tracking

Adverse events (AEs) and device deficiencies were documented and categorized in accordance with ISO14155:2020. These AEs were documented based on reports provided by the participants through email or on the exit survey. The investigators closely tracked the AEs and their resolution throughout the study. Each AE was categorized by type and seriousness according to the definitions provided in ISO14155. Whether an AE was related to the device or procedures was also distinguished. All available details for each AE were recorded in the participant case report forms, including relationship to the investigational device, severity (mild, moderate, or severe), onset date, resolution status, any action taken, and if there were any sequelae. For the causality assessment of all AEs, the MDCG 2020-10/1 guideline was followed. This guidance is specifically aimed at severe adverse events; however, it was extrapolated to all AEs for this study.

According to MDCG 2020-10/1, causal relatedness was defined as an AE associated with the investigational device beyond reasonable doubt. Probably device-related was defined as having a relationship with the use of the investigational device that seems relevant or the event cannot be reasonably explained by another cause. “Possibly device related” was defined as having a relationship with the use of the investigational device that was weak but cannot be ruled out completely. “Not device related” was defined as an event not having a temporal relationship with the device or not following a known response pattern to the device. The AEs were then further classified into mild, moderate, or severe categories. Mild severity AEs correspond to awareness of easily tolerated and mildly irritating signs or symptoms, with no or minimal loss of time from normal activities; these symptoms are transient and do not require therapy or a medical evaluation. Moderate cases are events that introduce a low level of inconvenience or concern to the participant and may interfere with daily activities; moderate experiences may cause some interference with functioning. Severe cases are events that substantially interrupt the participant’s normal daily activities and generally require systemic drug therapy or other treatment; these events are usually incapacitating.

### Statistical Analysis

The primary and secondary end points of the study are thoroughly described in the “Data Collection” section. These end points included pre- to posttreatment changes from baseline to the end of treatment at 4 weeks for the BAI as the primary end point and the BDI, PCL-5, and PSQI as secondary end points. Baseline scores were defined as the BAI, BDI, PCL-5, and PSQI scores on the first day of treatment, prior to the first treatment session. The within-group analyses were based on a per-protocol estimand and tested with paired 2-tailed *t* tests, where the normality assumption was confirmed with the Shapiro-Wilk test and α value was set to .05. The effect sizes reported in this paper are based on Cohen *d* and calculated as the mean score at the end of treatment minus the mean score at baseline, divided by the pooled SD for the 2 scores. The use of per-protocol estimand ensured that the changes in outcome measures within each treatment arm were reflective of scenarios where the participants used the treatment as directed and thus included only the participants who were compliant to treatment. The usage criteria for inclusion in the per-protocol analysis was set at 5-29 minutes of treatment per day 6-7 days per week across the intended 4-week treatment period. There were only 2 missing scores, 1 in week 2 and 1 in week 3. Because these data are a time series that exhibits a trend line and the number of missing values was very small, these were filled using a linear interpolation between the score from the previous week and the score from the following week. There was no missing baseline or final scores.

To determine the appropriate sample size a power analysis was performed assuming a dependent *t* test with a significance level of 5%, power of 80%, and moderate effect size of 0.6 between pairs. This gave us a necessary sample size of 25 participants. Accounting for a potential dropout rate of 20% gave us a target sample size of 30 participants. All analyses were performed using GraphPad Prism 10.3.0 (507; Dotmatics).

### Ethical Considerations

Ethical approval for this trial was obtained from the WIRB-Copernicus Group (WCG) institutional review board (reference no. 20233919), and the study was conducted in compliance with the principles outlined in the Declaration of Helsinki. Signed and documented informed consent was obtained from all participants prior to starting the study. For their time, participants were given a US $25.00 Amazon gift card.

## Results

### Study Participants

**Figure 3.**
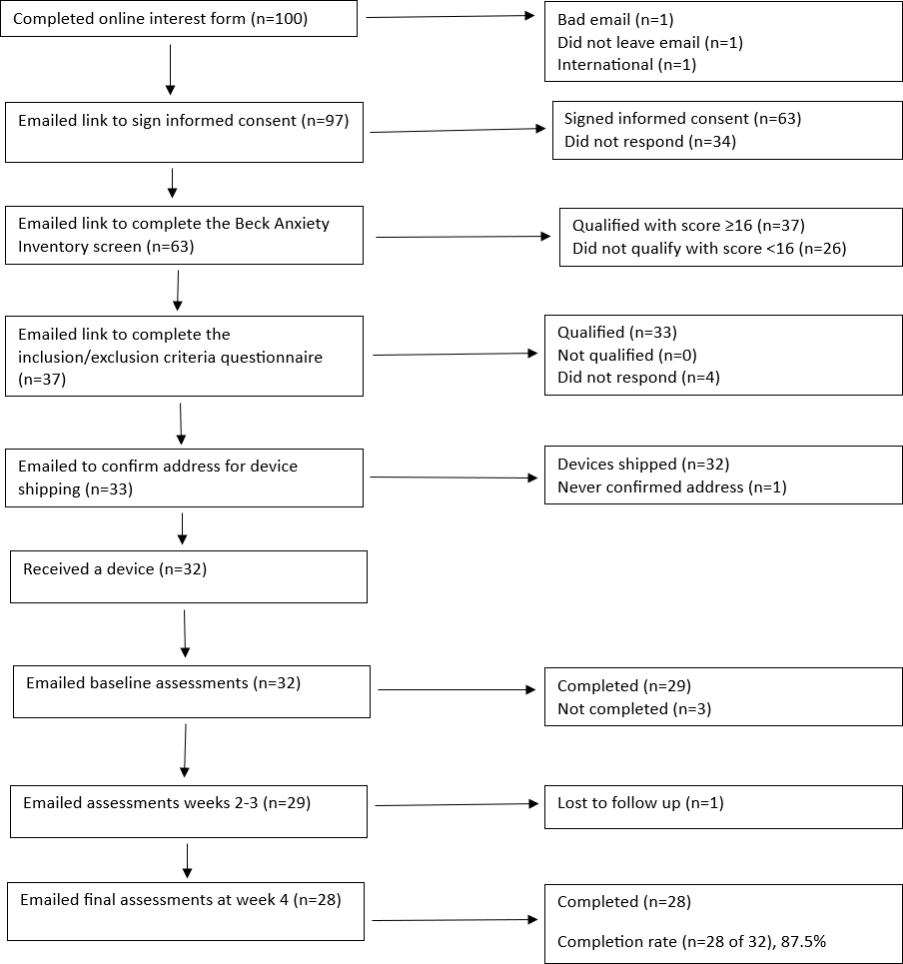
Flowchart of study participants through the trial.

Between October 22, 2023, and October 2, 2024, 100 individuals completed the web-based interest form (Figure 3). A total of 63 participants consented to the trial, with 26 of these not satisfying the criteria of having a BAI score of 16 or greater, 4 not responding to requests to complete the screening questionnaire, and 1 not responding to requests for confirmation of their shipping address. A total of 32 participants were shipped a device, with 3 of these not responding to requests to complete the baseline assessments and 1 participant failing to respond to requests to take the reassessments after week 2. In total, 28 participants completed all LIFU sessions and weekly assessments (28/32, 87.5%). Data for all 28 participants who completed the trial are included in the analysis.

The average age of the participants was 48.1 (SD 15.6) years. The group was heavily weighted toward women, with 22 women and 6 men. The National Institutes of Health reports that generalized anxiety affects approximately 2.7% of American adults, with women experiencing the disorder at a higher rate (3.4%) versus men (1.9%), making the fact that the sample contained a higher percentage of women a reflection of actual population distributions. The self-reported average duration of time suffering with anxiety was 16.5 (SD 11.8) years. There were also 8 participants currently receiving treatment for their anxiety and 20 who were not receiving any treatment.

### Beck Anxiety Inventory

**Figure 4.**
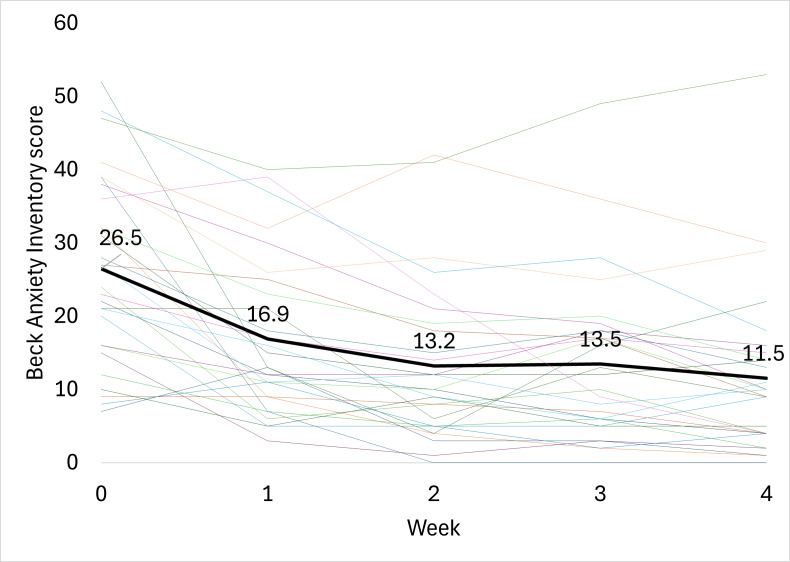
The progression of Beck Anxiety Inventory scores through 4 weeks of treatment with ZenBud. The thin lines represent each individual participant. The thick line represents the group mean.

**Figure 5.**
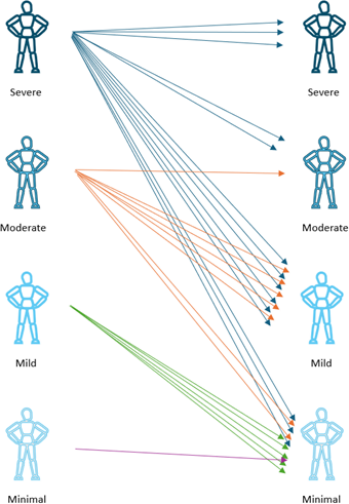
Categorical movement across degrees of severity based on the Beck Anxiety Inventory (BAI) definitions. At the start of the study, 20 participants had BAI scores in the moderate or severe anxiety ranges and only 6 participants had BAI scores in the mild or minimal severity ranges. After 4 weeks of using the ZenBud, 20 participants had BAI scores into the mild or minimal severity ranges, and only 6 participants had scores in the moderate or severe ranges.

After 4 weeks of treatment with the ZenBud, the average BAI score decreased by 14.9 (SD 10.6) points from 26.5 (SD 12.5) to 11.5 (SD 11.1) (Figure 4). This change in score was both statistically significant (*P*<.001, 2-tailed dependent *t* test) and clinically meaningful. While there is no consistently defined definition of clinical improvement for the BAI, based on the categorical definitions of severity for the scores, there was a great deal of progression into decreased severity levels of anxiety throughout the treatment period. As seen in Figure 5, at the start of the study, 22 participants had BAI scores in the moderate or severe anxiety ranges and only 6 participants had BAI scores in the mild or minimal severity ranges. After 4 weeks of using the ZenBud, 22 participants had BAI scores into the mild or minimal severity rages, and only 6 participants had scores in the moderate or severe ranges. In terms of Cohen *d*, the effect size was large at 1.06.

### Beck Depression Inventory

**Figure 6.**
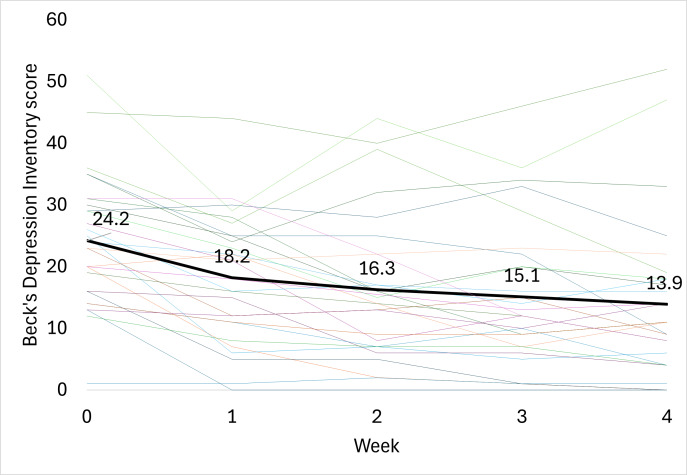
The progression of Beck Depression Inventory scores through 4 weeks of treatment with ZenBud. The thin lines represent each individual participant. The thick line represents the group mean.

After 4 weeks of treatment with the ZenBud, the average BDI score decreased by 10.3 (SD 7.8) points from 24.2 (SD 10.5) to 13.9 (SD 12.6) (Figure 6). Similar to results seen for the BAI, this change in score was both statistically significant (*P*<.001; 2-tailed dependent *t* test) and clinically meaningful. A 17% reduction in score on the BDI is considered clinically meaningful [3]. Based on this definition, as seen in Table 1, 71.4% (20/28) of participants demonstrated a clinically meaningful reduction in score by the end of the trial. In terms of Cohen *d*, the effect size was large at 0.81.

**Table 1.** The number of participants who experienced clinically significant reductions in Beck Depression Inventory score following 4 weeks of treatment with the ZenBud.

Degree of score change	Participants, n (%)
Clinical decrease	20 (71)
Nonclinical decrease	5 (18)
Nonclinical increase	3 (11)

### Post Traumatic Stress Disorder Checklist for the *DSM-V*

**Figure 7.**
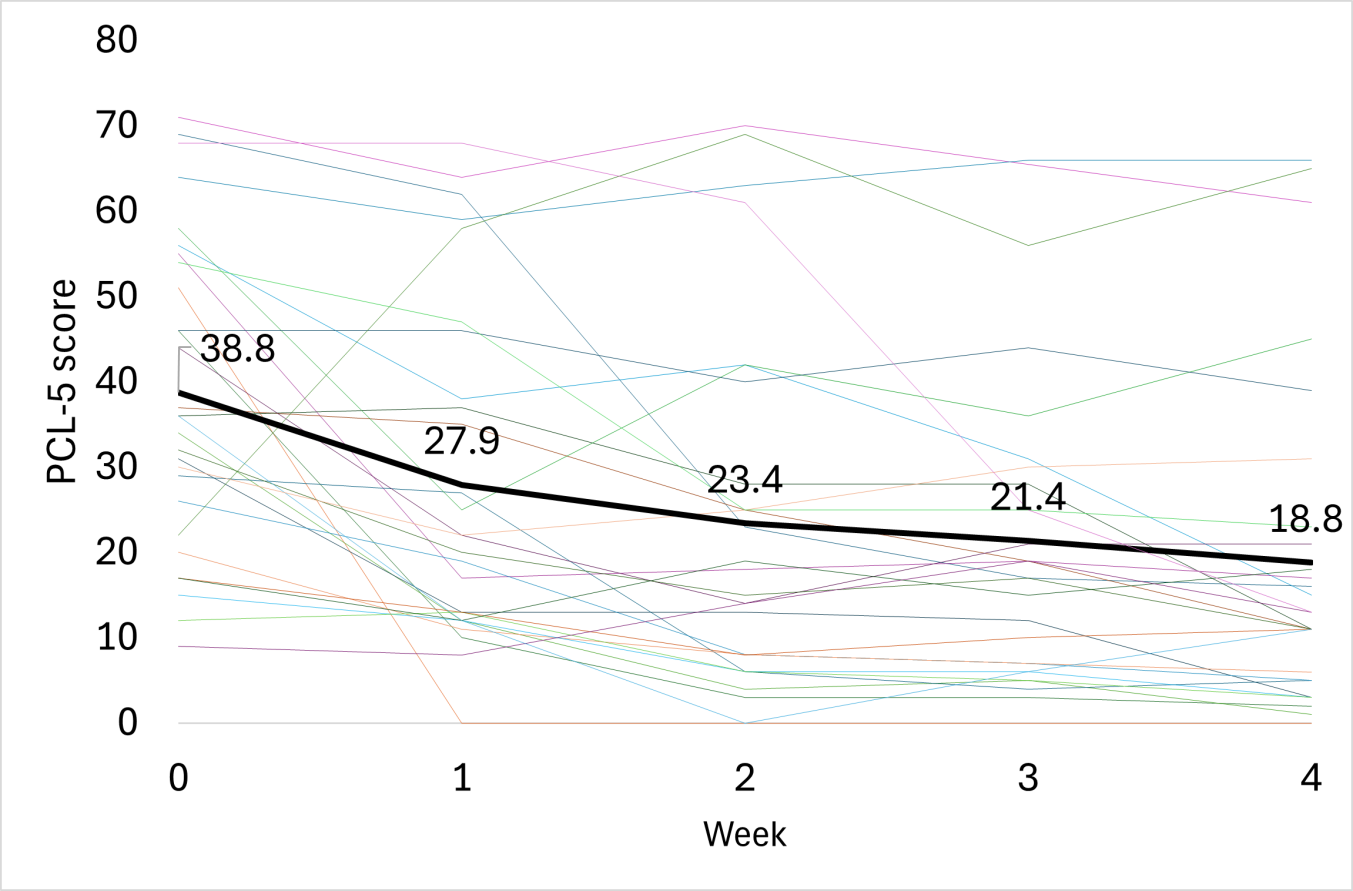
The progression of PCL-5 scores through 4 weeks of treatment with ZenBud. The thin lines represent each individual participant. The thick line represents the group mean.

After 4 weeks of treatment with the ZenBud, the average PCL-5 score decreased by 20.0 (SD 20.5) points from 38.8.8 (SD 18.0) to 18.8 (SD 18.9) (Figure 7). Similar to results seen for the BAI and BDI, this change in score was both statistically significant (*P*<.001; 2-tailed dependent *t* test) and clinically meaningful. A 10-point reduction in score on the PCL-5 is considered clinically meaningful [43,51]. Based on this definition, as seen in Table 2 , 71.4% (20/28) of participants demonstrated a clinically meaningful reduction in score by the end of the trial. In terms of Cohen *d*, the effect size was large at 0.94.

**Table 2.** The number of participants who experienced clinically significant reductions in PCL-5 score following 4 weeks of treatment with the ZenBud.

Degree of score change	Participants, n (%)
Clinical decrease	20 (71)
Nonclinical decrease	3 (11)
Nonclinical increase	4 (14)
Clinical increase	1 (4)

### Pittsburgh Sleep Quality Index

**Figure 8.**
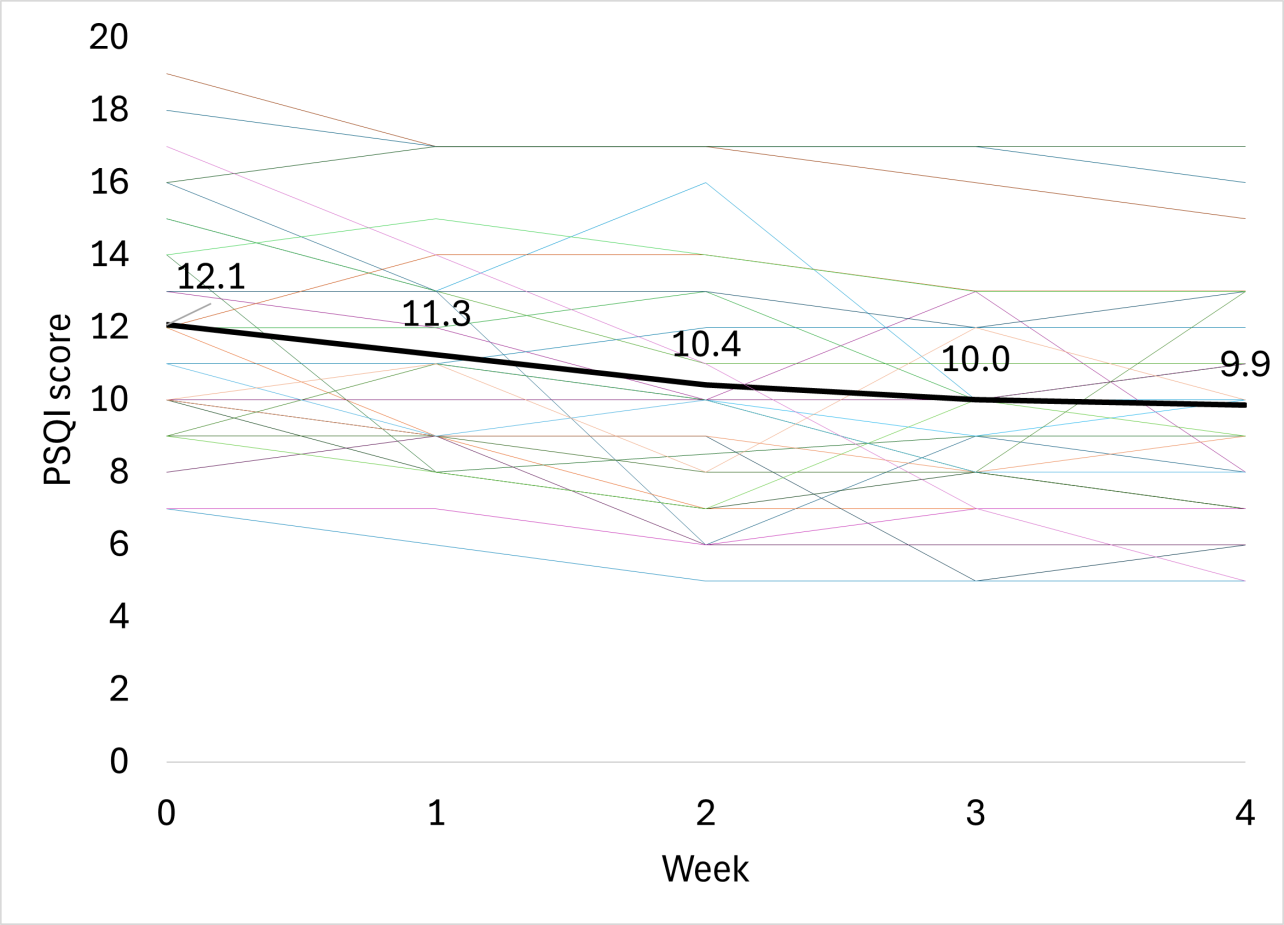
The progression of PSQI scores through 4 weeks of treatment with ZenBud. The thin lines represent each individual participant. The thick line represents the group mean. PSQI: Pittsburgh Sleep Quality Index.

After 4 weeks of treatment with the ZenBud, the average PSQI score decreased by 2.2 (SD 3.1) points from 12.1 (SD 3.2) to 9.9 (SD 3.2) (Figure 8). While this change in score was statistically significant (*P*=.001; 2-tailed dependent *t* test), it was not clinically meaningful. In terms of Cohen *d*, the effect size was medium at 0.65.

### Satisfaction and Acceptability

**Figure 9.**
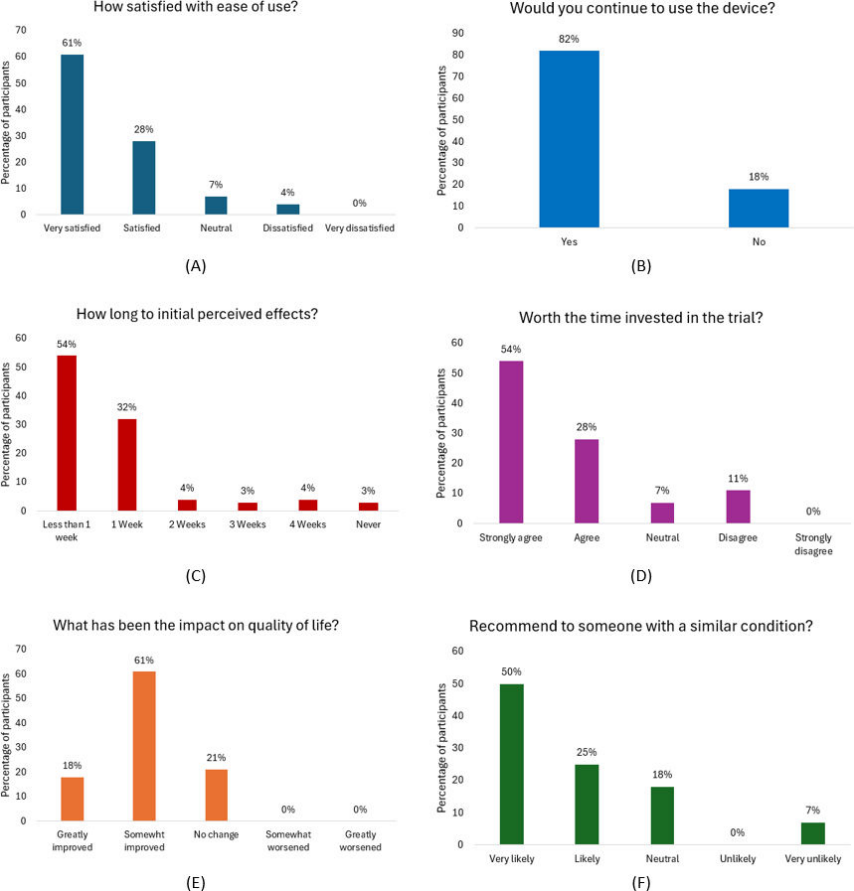
Results of the exit survey. (A) Responses of the participants when asked “How satisfied were you with the ease of using the device?” (B) Responses of the participants when asked “Would you continue using this device for treatment?” (C) Responses of the participants when asked “How quickly did you feel the effects of the ZenBud device during your trial?” (D) Responses of the participants when asked “Do you feel the device was worth the time invested in the trial?” (E) Responses of the participants when asked “How did the device impact your overall quality of life?” (F) Responses of the participants when asked “How likely are you to recommend this device to others with similar conditions?.”

After the final treatment and assessment, battery participants completed an exit survey asking questions regarding satisfaction with the treatment, acceptability, and quality-of-life impact. When asked about satisfaction with ease of use, 89.3% (25/28) of participants responded with very satisfied or satisfied (Figure 9A). In addition, 82.1% (23/28) of participants reported that they would continue using the device if offered the opportunity (Figure 9B). When asked whether the treatment was worth the time invested in the trial, 82.1% (23/28) of participants strongly agreed or agreed that the time invested was worth it (Figure 9D). When asked about the impact on quality of life, 78.6% (22/28) of participants reported that the treatment somewhat or greatly impacted their quality of life (Figure 9E). When asked how long it took to feel initial effects, 53.6% 15/28) of participants noticed effects in less than 1 week and 32.1% (9/28) felt initial effects by 1 week (Figure 9C). When asked whether they would recommend the treatment to someone with a similar condition, 75.0% (21/28) of participants responded with very likely or likely (Figure 9F).

### Adverse Events

Only 1 AE was reported throughout the duration of the trial. On the exit survey following completion of the 4 weeks of treatment, 1 participant reported that the treatment would make them feel jittery for a short period of time afterward. This effect was short-lived and classified as a mild AE that was probably device related. The participant reported that this side effect was not enough of an effect to make them stop treatment or drop out of the study. Overall, the high satisfaction rates as described in the “Satisfaction and Acceptability” section combined with the low rate of AE support a strong benefit-to-risk profile for the ZenBud. However, this study was done with a small sample size and these results need to be further validated with a larger sample size.

## Discussion

### Principal Findings

The main objective of this study was to provide preliminary evidence of the efficacy, safety, and usability of the ZenBud for treating symptoms of anxiety in humans. Overall, the study represents one of the first clinical trials supporting the safety, patient tolerability, and efficacy of using LIFU to the auricular branch of the vagus nerve for the treatment of anxiety symptoms.

Among the 28 participants, 92.9% (26/28) demonstrated improvements in anxiety symptoms, 89.3% (25/28) demonstrated improvements in depression symptoms, 82.1% (23/28) demonstrated a reduction in symptoms of PTSD, and 65.5% (18/28) demonstrated improvements in sleep quality after 4 weeks of treatment. The average score reduction on the BAI was clinically meaningful at 14.9 points (SD 10.6, *P*<.001; 2-tailed dependent *t* test), reflecting a general movement from severe anxiety symptoms to mild [35,36]. The average score reduction on the BDI was clinically meaningful at 10.3 points (SD 7.8, *P*<.001; 2-tailed dependent *t* test), which was a 42.6% decrease in score, far greater than the 17% clinically meaningful threshold [3]. The average score reduction on the PCL-5 was clinically meaningful at 20.0 points (SD 20.5, *P*<.001; 2-tailed dependent *t* test) [43]. It is also noteworthy to mention that the PCL-5 is commonly used to determine whether an individual meets a provisional diagnosis of PTSD and requires further assessment to confirm the diagnosis. The cutoff score for meeting the criteria for a provisional PTSD diagnosis is 31‐33. Based on using a cutoff score of 32, at the start of the study 18 participants exceeded the threshold score for a provisional PTSD diagnosis. Upon completion of the study, 14 of these participants (77.8%) had dropped their score below the threshold score of 32 and no longer met the requirements for a provisional PTSD diagnosis. The average score reduction on the PSQI was 2.2 (SD 3.1, *P*=.001; 2-tailed dependent *t* test) which, while statistically significant, was not clinically meaningful, indicating that the improvements in anxiety, depression, and PTSD symptoms did not carry over into improved sleep quality. The effect sizes were also large for the BAI (Cohen *d*=1.06), BDI (Cohen *d*=0.81), and PCL-5 (Cohen *d*=0.94) indicating that the observed score improvements were substantial enough to have a meaningful impact beyond just statistical significance.

The extent of improvement in anxiety, depression, and PTSD observed in this study is comparable with the clinically meaningful results reported in other clinical trials featuring noninvasive VNS as a treatment intervention. Srinivasan et al [52] conducted a randomized controlled trial of taVNS with 60 retired schoolteachers who had been diagnosed with anxiety during the COVID-19 pandemic. The participants did 30-minute sessions 4 times per week (16 total sessions) and demonstrated significantly greater reductions in Generalized Anxiety Disorder-7 (GAD-7) scores and salivary cortisol levels compared with control group participants. Zhang et al [53] investigated the effect of taVNS on anxiety symptoms and neural functioning in 30 individuals with Parkinson disease and anxiety compared with 30 controls with no anxiety. They treated patients with Parkinson disease with taVNS for 2 weeks and measured progress using the HAM-A and nerve activation in the bilateral prefrontal cortex during a verbal fluency task. After 2 weeks of taVNS treatment, the group demonstrated a significant decrease in HAM-A scores (*P*<.001) and increased activation of the left triangle portion of the inferior frontal gyrus. Ferreira et al [54] treated college students with chronic anxiety with a week of taVNS. Immediately postintervention and 2 weeks postintervention the students demonstrated substantial reductions in pain perception, Beck Anxiety Inventory scores, and masseter activation. Rong et al [55] treated 91 patients with mild to moderate depression with taVNS for 30 minutes twice a day for 12 weeks. Upon completion of treatment the average reduction in score in the 24-item Hamilton Depression Rating Scale (HAM-D-24) was both statistically significant and clinically meaningful, the responder rate was 80%, and the remission rate was 39%. In our study, we saw similar results in only 4 weeks, making an investigation into longer treatment periods with LIFU an important area of future research.

The results of this study are also consistent with the results of studies investigating the use of transcranial focused ultrasound (tfUS) targeting the amygdala for the treatment of generalized anxiety disorders. Mahdavi et al [56] recruited 25 participants with treatment-refractory generalized anxiety disorder and treated them with tfUS targeting the right amygdala for 8 weekly 10-minute sessions. The results showed an average reduction in BAI score of 12.88 (SD 10.42) points and an average reduction in HAM-A scores of −12.64 (SD 12.51). Chou et al [57] recruited 30 healthy individuals and compared activation of the amygdala, hippocampus, and dorsal anterior cingulate cortex during a fear task after treating them with active or sham tfUS targeting the left amygdala. They found decreased activation of the amygdala (*P*=.04), hippocampus (*P*=.05), and dorsal anterior cingulate (*P*=.02) in the active tfUS group when compared with the sham. They also found decreased amygdala-insula (*P*=.03) and amygdala-hippocampal (*P*=.01) resting state functional connectivity and increased amygdala-ventromedial prefrontal cortex (*P*=.05) resting state functional connectivity.

### Limitations

While the results of this study are optimistic, this study was preliminary and suffers from several limitations. This study did not feature a control group, making it impossible to quantify the possible impact of a placebo effect or distinguish the specific effects of the ZenBud device from other factors that may have influenced the results. The lack of a control group also limits the ability to directly compare the efficacy of the ZenBud with other interventions. Other than participant reports, there was also no objective way of determining the exact amount of time the device was used by each participant. While the majority of participants were not receiving treatment during the study, there was no control over concurrent therapeutic modalities participants were receiving. The lack of control for these additional therapies may have influenced the results, making it difficult to attribute the observed effects exclusively to the ZenBud device. Further research with larger sample sizes, control groups, control over concurrent treatment modalities, and physiological measurements needs to be done to validate these findings and further negate the possibility of placebo effects.

### Conclusions

This preliminary study provided justification for further research into the efficacy, safety, and feasibility of using LIFU to modulate the auricular branch of the vagus nerve and reduce the symptoms of anxiety, depression, and PTSD. Given the wide prevalence of anxiety disorders, depression, and PTSD, and the shortfalls of current treatment options, this novel treatment approach has potential to meaningfully improve patient outcomes and continued research is warranted.

## Supplementary material

10.2196/69770 Multimedia Appendix 1 ZenBud user manual.

## Abbreviations

AE: adverse event
BAI: Beck Anxiety Inventory
BDI: Beck Depression Inventory
*DSM-V*: *Diagnostic and Statistical Manual of Mental Disorders* (Fifth Edition)
HAM-A: Hamilton Anxiety Rating Scale
LIFU: low-intensity focused ultrasound
PSQI: Pittsburgh Sleep Quality Index
PTSD: posttraumatic stress disorder
SNS: sympathetic nervous system
taVNS: transcutaneous auricular vagal nerve stimulation
tfUS: transcranial focused ultrasound
VNS: vagus nerve stimulation

## References

[1] Penninx BW, Pine DS, Holmes EA, Reif A (2021). Anxiety disorders. Lancet.

[2] Chand SP, Marwaha R (2023). StatPearls. Anxiety.

[3] Button KS, Kounali D, Thomas L (2015). Minimal clinically important difference on the Beck Depression Inventory—II according to the patient’s perspective. Psychol Med.

[4] Tasker JG, Joëls M, Russell J, Shipston M (2015). Neuroendocrinology of Stress.

[5] Holwerda SW, Luehrs RE, Gremaud AL (2018). Relative burst amplitude of muscle sympathetic nerve activity is an indicator of altered sympathetic outflow in chronic anxiety. J Neurophysiol.

[6] Giulio P (2021). Maladaptive stress: theoretical, neurobiological and clinical profiles. Arch Depress Anxiety.

[7] Ströhle A, Gensichen J, Domschke K (2018). The diagnosis and treatment of anxiety disorders. Dtsch Arztebl Int.

[8] Stockings EA, Degenhardt L, Dobbins T (2016). Preventing depression and anxiety in young people: a review of the joint efficacy of universal, selective and indicated prevention. Psychol Med.

[9] Fischer R, Bortolini T, Karl JA (2020). Rapid review and meta-meta-analysis of self-guided interventions to address anxiety, depression, and stress during COVID-19 social distancing. Front Psychol.

[10] Haller H, Breilmann P, Schröter M, Dobos G, Cramer H (2021). A systematic review and meta-analysis of acceptance- and mindfulness-based interventions for DSM-5 anxiety disorders. Sci Rep.

[11] Chi D, Zhang Y, Zhou D, Xu G, Bian G (2022). The effectiveness and associated factors of online psychotherapy on COVID-19 related distress: a systematic review and meta-analysis. Front Psychol.

[12] Moghimi E, Stephenson C, Agarwal A (2023). Efficacy of an electronic cognitive behavioral therapy program delivered via the online psychotherapy tool for depression and anxiety related to the COVID-19 pandemic: pre-post pilot study. JMIR Ment Health.

[13] Garakani A, Murrough JW, Freire RC (2020). Pharmacotherapy of anxiety disorders: current and emerging treatment options. Front Psychiatry.

[14] Baek H, Pahk KJ, Kim H (2017). A review of low-intensity focused ultrasound for neuromodulation. Biomed Eng Lett.

[15] Collins MN, Mesce KA (2022). A review of the bioeffects of low-intensity focused ultrasound and the benefits of A cellular approach. Front Physiol.

[16] Feng B, Chen L, Ilham SJ (2019). A review on ultrasonic neuromodulation of the peripheral nervous system: enhanced or suppressed activities?. Appl Sci (Basel).

[17] Johnson RL, Wilson CG (2018). A review of vagus nerve stimulation as A therapeutic intervention. J Inflamm Res.

[18] Martin EI, Ressler KJ, Binder E, Nemeroff CB (2009). The neurobiology of anxiety disorders: brain imaging, genetics, and psychoneuroendocrinology. Psychiatr Clin North Am.

[19] Kaniusas E, Kampusch S, Tittgemeyer M (2019). Current directions in the auricular vagus nerve stimulation I—a physiological perspective. Front Neurosci.

[20] Butt MF, Albusoda A, Farmer AD, Aziz Q (2020). The anatomical basis for transcutaneous auricular vagus nerve stimulation. J Anat.

[21] Breit S, Kupferberg A, Rogler G, Hasler G (2018). Vagus nerve as modulator of the brain-gut axis in psychiatric and inflammatory disorders. Front Psychiatry.

[22] Noble LJ, Gonzalez IJ, Meruva VB (2017). Effects of vagus nerve stimulation on extinction of conditioned fear and post-traumatic stress disorder symptoms in rats. Transl Psychiatry.

[23] George MS, Ward HE, Ninan PT (2008). A pilot study of vagus nerve stimulation (VNS) for treatment-resistant anxiety disorders. Brain Stimul.

[24] Gurel NZ, Wittbrodt MT, Jung H (2020). Transcutaneous cervical vagal nerve stimulation reduces sympathetic responses to stress in posttraumatic stress disorder: a double-blind, randomized, sham controlled trial. Neurobiol Stress.

[25] Badran BW, Huffman SM, Dancy M (2022). A pilot randomized controlled trial of supervised, at-home, self-administered transcutaneous auricular vagus nerve stimulation (taVNS) to manage long COVID symptoms. Res Sq.

[26] Wittbrodt MT, Gurel NZ, Nye JA (2021). Noninvasive cervical vagal nerve stimulation alters brain activity during traumatic stress in individuals with posttraumatic stress disorder. Psychosom Med.

[27] Lamb DG, Porges EC, Lewis GF, Williamson JB (2017). Non-invasive vagal nerve stimulation effects on hyperarousal and autonomic state in patients with posttraumatic stress disorder and history of mild traumatic brain injury: preliminary evidence. Front Med (Lausanne).

[28] Bremner J, Gurel N, Wittbrodt M (2019). Non-invasive vagal nerve stimulation paired with stress exposure in posttraumatic stress disorder (PTSD). Brain Stimul.

[29] Riis T, Kubanek J (2022). Effective ultrasonic stimulation in human peripheral nervous system. IEEE Trans Biomed Eng.

[30] Juan EJ, González R, Albors G, Ward MP, Irazoqui P (2014). Vagus nerve modulation using focused pulsed ultrasound: potential applications and preliminary observations in a rat. Int J Imaging Syst Technol.

[31] Cotero V, Fan Y, Tsaava T (2019). Noninvasive sub-organ ultrasound stimulation for targeted neuromodulation. Nat Commun.

[32] Bentley KH, Bernstein EE, Wallace B, Mischoulon D (2021). Treatment for anxiety and comorbid depressive disorders: transdiagnostic cognitive-behavioral strategies. Psychiatr Ann.

[33] Williamson JB, Jaffee MS, Jorge RE (2021). Posttraumatic stress disorder and anxiety-related conditions. Continuum (Minneap Minn).

[34] Fydrich T, Dowdall D, Chambless DL (1992). Reliability and validity of the Beck Anxiety Inventory. J Anxiety Disord.

[35] Beck AT, Epstein N, Brown G, Steer RA (1988). An inventory for measuring clinical anxiety: psychometric properties. J Consult Clin Psychol.

[36] Beck AT, Steer RA (1990). Manual for the Beck Anxiety Inventory.

[37] Bardhoshi G, Duncan K, Erford BT (2016). Psychometric meta‐analysis of the English version of the Beck Anxiety Inventory. Jour of Counseling & Develop.

[38] Beck AT, Steer RA, Carbin MG (1988). Psychometric properties of the Beck Depression Inventory: twenty-five years of evaluation. Clin Psychol Rev.

[39] Maletic V, Robinson M, Oakes T, Iyengar S, Ball SG, Russell J (2007). Neurobiology of depression: an integrated view of key findings. Int J Clin Pract.

[40] Beck AT, Ward CH, Mendelson M, Mock J, Erbaugh J (1961). An inventory for measuring depression. Arch Gen Psychiatry.

[41] Beck AT, Steer RA, Ball R, Ranieri WF (1996). Comparison of Beck Depression Inventories -IA and -II in psychiatric outpatients. J Pers Assess.

[42] Wang YP, Gorenstein C (2013). Psychometric properties of the Beck Depression Inventory—II: a comprehensive review. Braz J Psychiatry.

[43] Blanchard BE, Johnson M, Campbell SB (2023). Minimal important difference metrics and test-retest reliability of the PTSD Checklist for DSM-5 with a primary care sample. J Trauma Stress.

[44] Blevins CA, Weathers FW, Davis MT, Witte TK, Domino JL (2015). The Posttraumatic Stress Disorder Checklist for DSM-5 (PCL-5): development and initial psychometric evaluation. J Trauma Stress.

[45] Forkus SR, Raudales AM, Rafiuddin HS, Weiss NH, Messman BA, Contractor AA (2023). The Posttraumatic Stress Disorder (PTSD) Checklist for DSM-5: a systematic review of existing psychometric evidence. Clin Psychol (New York).

[46] Cox RC, Olatunji BO (2016). A systematic review of sleep disturbance in anxiety and related disorders. J Anxiety Disord.

[47] K.p S, Joseph N, Sreenivasan GK (2021). Sleep quality and anxiety level among college students. Int J Res Rev.

[48] Buysse DJ, Reynolds CF, Monk TH, Berman SR, Kupfer DJ (1989). The Pittsburgh Sleep Quality Index: a new instrument for psychiatric practice and research. Psychiatry Res.

[49] Mollayeva T, Thurairajah P, Burton K, Mollayeva S, Shapiro CM, Colantonio A (2016). The Pittsburgh Sleep Quality Index as a screening tool for sleep dysfunction in clinical and non-clinical samples: a systematic review and meta-analysis. Sleep Med Rev.

[50] Fabbri M, Beracci A, Martoni M, Meneo D, Tonetti L, Natale V (2021). Measuring subjective sleep quality: a review. Int J Environ Res Public Health.

[51] Held P, Smith DL, Bagley JM (2021). Treatment response trajectories in a three-week CPT-based intensive treatment for veterans with PTSD. J Psychiatr Res.

[52] Srinivasan V, Ruthuvalan V, Raja S (2024). Efficacy of vagal nerve stimulation on anxiety among elderly retired teachers during COVID-19 pandemic. Work.

[53] Zhang H, Shan A di, Wan CH (2024). Transcutaneous auricular vagus nerve stimulation improves anxiety symptoms and cortical activity during verbal fluency task in Parkinson’s disease with anxiety. J Affect Disord.

[54] Ferreira LMA, Brites R, Fraião G (2024). Transcutaneous auricular vagus nerve stimulation modulates masseter muscle activity, pain perception, and anxiety levels in university students: a double-blind, randomized, controlled clinical trial. Front Integr Neurosci.

[55] Rong P, Liu J, Wang L (2016). Effect of transcutaneous auricular vagus nerve stimulation on major depressive disorder: a nonrandomized controlled pilot study. J Affect Disord.

[56] Mahdavi KD, Jordan SE, Jordan KG (2023). A pilot study of low-intensity focused ultrasound for treatment-resistant generalized anxiety disorder. J Psychiatr Res.

[57] Chou T, Deckersbach T, Guerin B (2024). Transcranial focused ultrasound of the amygdala modulates fear network activation and connectivity. Brain Stimul.

