# Supplementary material for: Reduction of Anxiety-Related Symptoms Using Low-Intensity Ultrasound Neuromodulation on the Auricular Branch of the Vagus Nerve: Preliminary Study
Source: JMIR Neurotechnol. 2025 May 1;4:e69770. doi: 10.2196/69770 (PMC12671299; doi:10.2196/69770)
Supplement: Multimedia Appendix 1 [file neuro-v4-e69770-s001.pdf]

ZenBud Headset User Manual

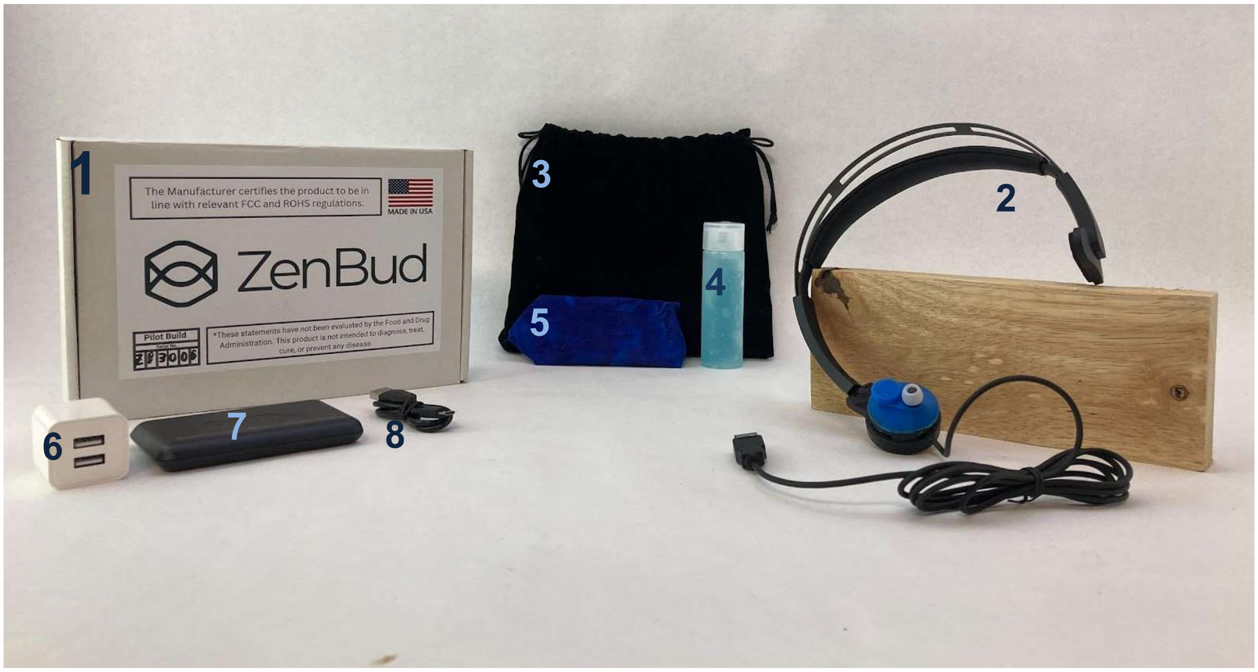

Unboxing

In your ZenBud Headset package, you will find:

1. ZenBud Box
2. ZenBud Headset
3. Black carry bag
4. Aquasonic Gel
5. Hand-sewn Cleaning cloth
6. USB wall block (US configuration, for charging battery pack)
7. ONN™ USB battery pack (for portable use)
8. USB cord (for charging battery pack)

Welcome to ZenBud

We thank you for choosing the ZenBud headset, a revolutionary device designed to bring a new level of relaxation and mindfulness into your life. Your trust in our product is deeply appreciated, and we are confident that the ZenBud will exceed your expectations.

This user manual has been designed to guide you through every aspect of your ZenBud experience, from initial setup to regular maintenance. It is comprehensive and easy to understand, written with both first-time and seasoned users in mind.

*These statements have not been evaluated by the Food and Drug Administration. This product is not intended to diagnose, treat, cure, or prevent any diseases.

In this manual, you will find:

1. **Unboxing**: Details of what is included with your ZenBud headset.

2. **Suggested Use**: Guidelines for optimal use, including the recommended duration of sessions.

3. **Putting it On and Using**: Step-by-step instructions on how to correctly wear your ZenBud headset, with particular attention to achieving the correct placement for optimal function.

4. **Powering the ZenBud**: Information on how to power your ZenBud headset and how to identify when it is functioning correctly.

5. **Tips and Tricks**: Additional advice to enhance your ZenBud experience, including how to adjust the headset for ultimate comfort and effectiveness.

6. **Cleaning and Storing**: Proper procedures for cleaning and storing your ZenBud headset to ensure its longevity.

7. **Help and Support**: Our contact information for when you need assistance or have questions about your ZenBud headset.

We hope that this user manual serves as a valuable resource in your journey with the ZenBud headset. Remember, our dedicated support team is always ready to assist should you need any help. We are committed to ensuring that your ZenBud experience is as pleasant and beneficial as possible.

If you have any questions or experience any discomfort with use, please reach out to Izzy Kohler, PhD, at izzy@neurgear.com or (585) 943-0580.

Once again, thank you for choosing ZenBud.

*These statements have not been evaluated by the Food and Drug Administration. This product is not intended to diagnose, treat, cure, or prevent any diseases.

ZenBud

Suggested Use

The ZenBud headset is designed to deliver the optimal experience in 5-minute sessions. However, the usage can be extended if recommended by a trained professional.

1. Prior to use, ensure that the ZenBud headset, Aquasonic Gel, and a charged USB battery pack are within easy reach. Then find a comfortable chair, mat, or location to begin stimulation.

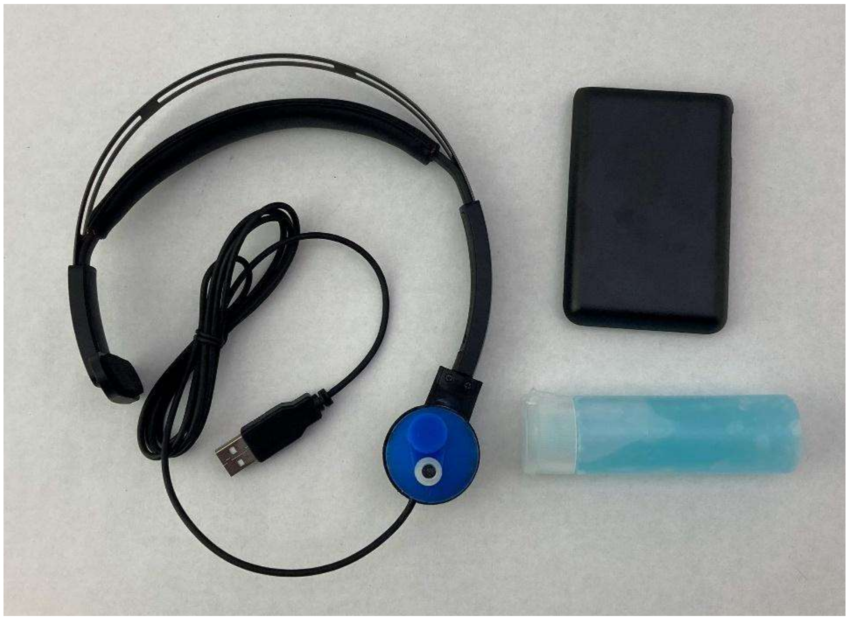

2. Apply a pea-sized amount of the Aquasonic Gel to the blue circular pad above the earbud.

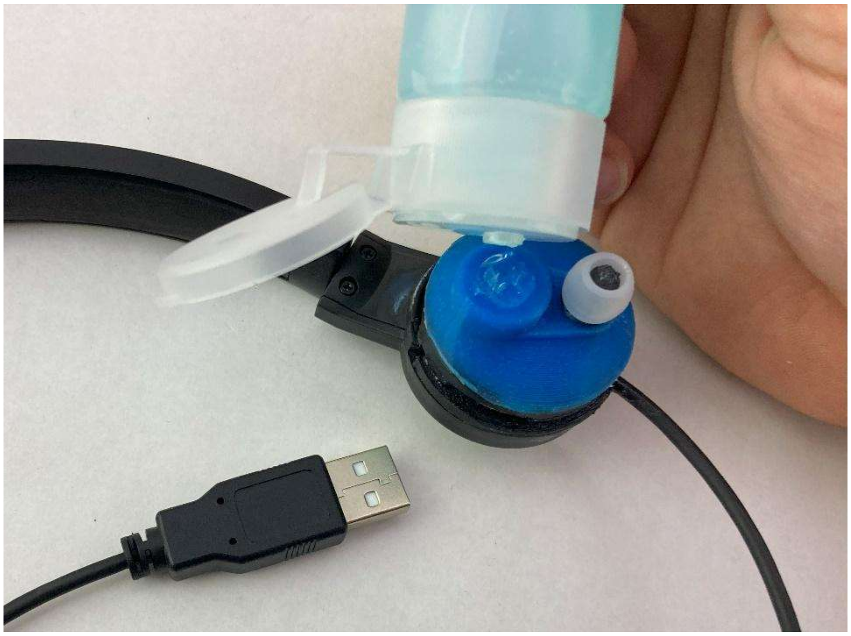

*These statements have not been evaluated by the Food and Drug Administration. This product is not intended to diagnose, treat, cure, or prevent any diseases.

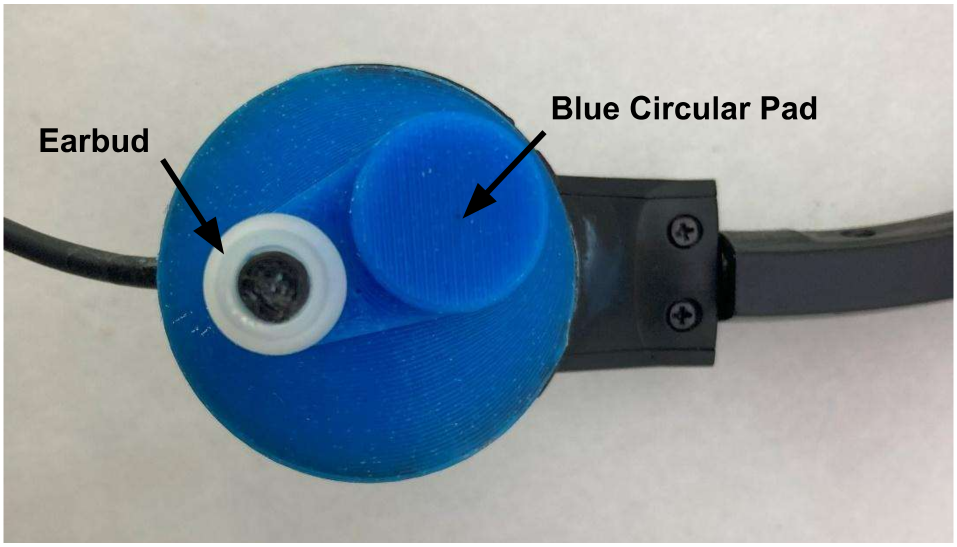

3. Gently insert the earbud into your right ear. It should fit securely and comfortably.

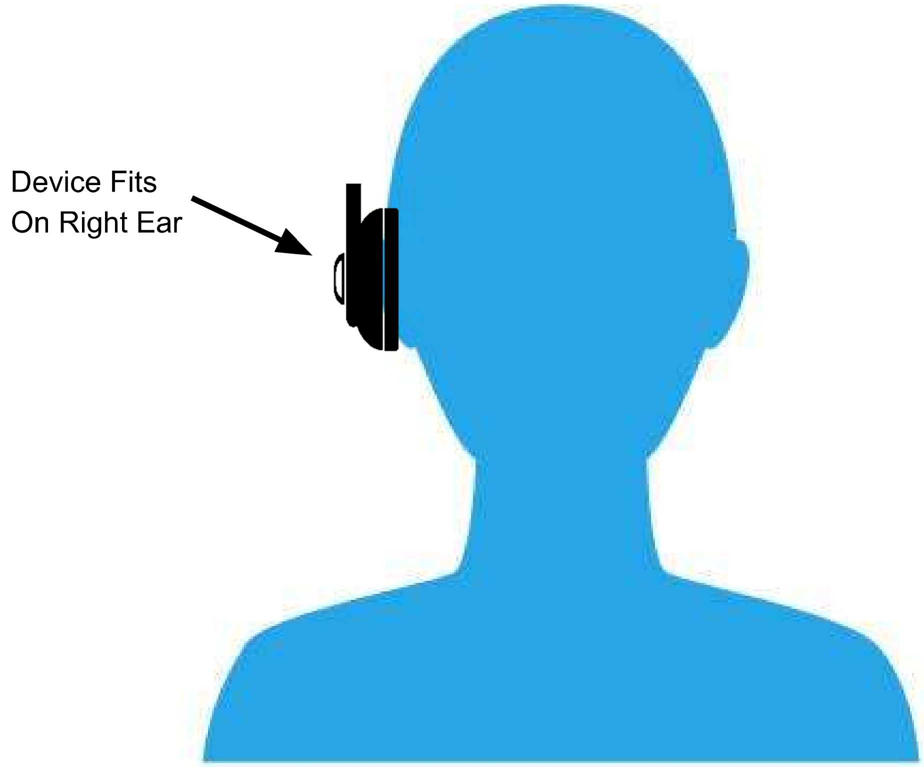

4. Press the gelled blue circular pad against your skin, right above your ear canal. Ensure good contact as poor contact will lead to a worse ZenBud experience.

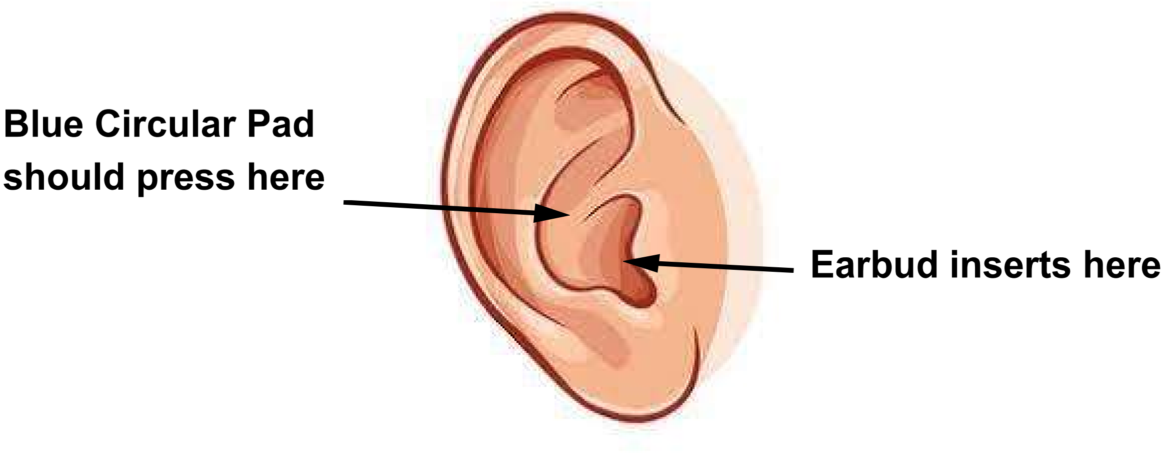

5. When you're ready to begin stimulation, plug the USB cable into the ONN™ battery pack or any compatible USB port.

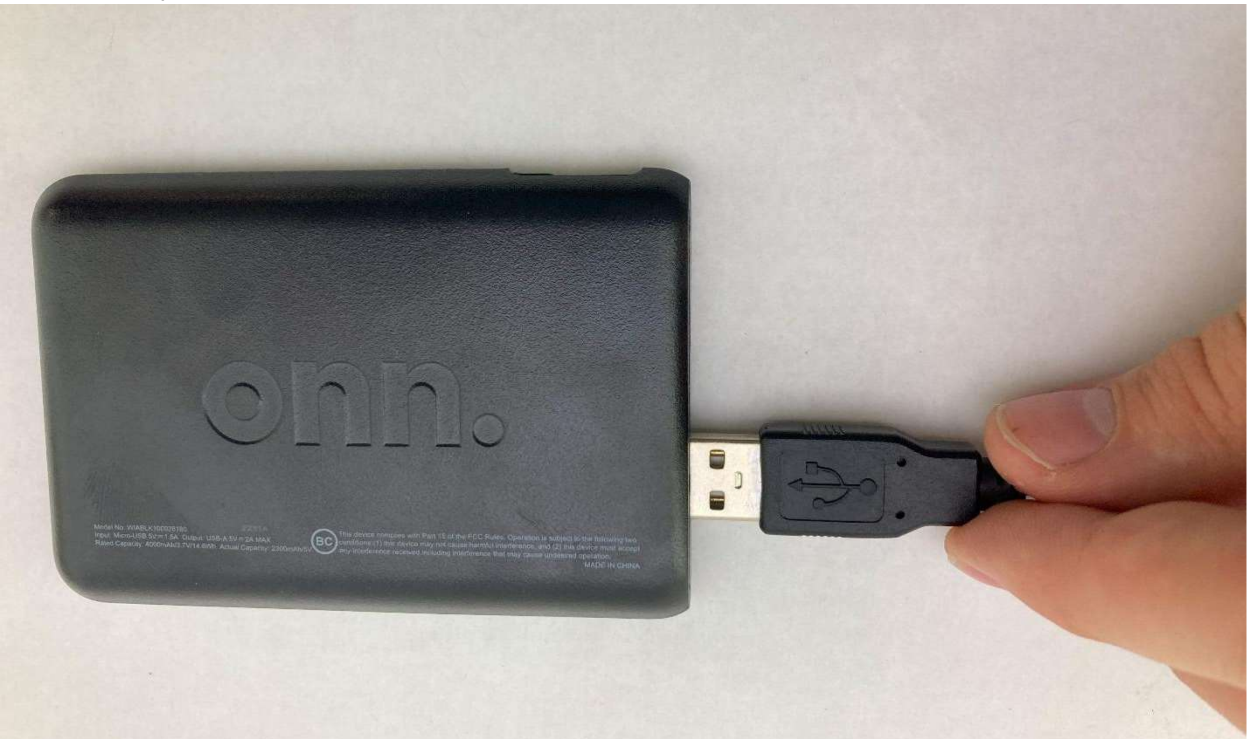

6. As soon as the ZenBud is connected to a power source, it will start to function. A low humming noise indicates that the device is powered and working. Enjoy your stimulation session!

*These statements have not been evaluated by the Food and Drug Administration. This product is not intended to diagnose, treat, cure, or prevent any diseases.

ZenBud

Tips and Tricks

To get the most out of your ZenBud experience:

1. Ensure you are relaxed and maintain deep, steady breathing during the session.
2. For optimal comfort, adjust the ZenBud so that the blue pad is firmly pressed against your ear. The adjustable strap should accommodate most head shapes comfortably.

Cleaning and Storing

After each use, follow these steps to clean and store the ZenBud headset:

1. Clean any remaining Aquasonic gel from your ear either using the provided cloth or a tissue.
2. Dampen the provided cloth and then clean the earbud and blue circular pad.
3. Store the ZenBud headset in its black carry bag and keep it in a cool, dry place when not in use.

Help and Support

If you encounter any problems or need assistance with use:

1. Refer to this user manual for helpful tips.
2. For further assistance, contact our support team via email at info@zenbud.health.

Thank you for choosing the ZenBud Headset. We hope you enjoy this revolutionary device as much as we enjoyed creating it for you.

*These statements have not been evaluated by the Food and Drug Administration. This product is not intended to diagnose, treat, cure, or prevent any diseases.

ZenBud

Proper Placement and Adjustment of the ZenBud Headset

Ensuring the correct placement and adjustment of the ZenBud headset is crucial for its optimal functioning and for you to get the most out of your sessions. Here are some further the detailed steps on how to properly position and secure the ZenBud headset:

1. **Insert the Earbud**: Gently insert the earbud into your ear. It should fit snugly without causing any discomfort.

2. **Apply Gel to the Blue Circular Pad**: Apply a pea sized amount of the included Aquasonic Gel to the blue part of the device. This part is located directly above the earbud.

3. **Position the Blue Circular Pad**: The lubricated blue part should be pressed against your skin, just above your ear canal. This location corresponds to the crux of helix and the innervated region of the auricular branch of the Vagus Nerve.

4. **Adjust Until You Feel Pressure**: Begin adjusting the ZenBud headset until you feel a moderate pressure just above your ear canal, where the blue circular pad is positioned. This pressure should be comfortable and not cause any pain or significant discomfort. The aim is to create a firm contact between the headset and your skin.

5. **Check the Device Operation**: Once properly positioned, your ZenBud headset should start functioning when connected to a power source. If you do not feel a low humming noise or do not experience the intended effects of the ZenBud, it could be due to improper placement.

If you don't feel the pressure above your ear canal or if the device is not functioning as intended, please try repositioning the ZenBud. You may need to move it slightly up, down, forward, or backward until you find the spot where you can feel the pressure and the device operates optimally.

Remember, everyone's anatomy is different, so what works best for you may not be the exact positioning as for someone else. Be patient and adjust the device as needed. With consistent use, finding the optimal placement will become quicker and easier.

Should you continue to have difficulty with proper placement or operation of the ZenBud headset, please don't hesitate to reach out to our support team at info@zenbud.health. We're here to help ensure your experience with ZenBud is as beneficial and comfortable as possible.

*These statements have not been evaluated by the Food and Drug Administration. This product is not intended to diagnose, treat, cure, or prevent any diseases.
